# Acute Kidney Injury in Childhood Nephrotic Syndrome: From Hemodynamic Vulnerability and Tubular Stress Toward Biomarker-Guided Management

**DOI:** 10.3390/medicina62101979

**Published:** 2026-10-13

**Authors:** John Dotis, Nikoleta Printza

**Affiliations:** 1 Third Department of Pediatrics, Hippokration Hospital, Aristotle University of Thessaloniki, 54642 Thessaloniki, Greece; yandot@auth.gr; 2 First Department of Pediatrics, Hippokration Hospital, Aristotle University of Thessaloniki, 54642 Thessaloniki, Greece

**Keywords:** acute kidney injury, nephrotic syndrome, children, volume status, tubular injury, biomarkers, phenotype-guided management

## Abstract

Acute kidney injury (AKI) is a clinically important, mechanistically heterogeneous complication of childhood nephrotic syndrome. Similar increases in serum creatinine may reflect reduced effective arterial volume, extracellular volume expansion with pressure-associated dysfunction, acute tubular stress or injury, infection or sepsis, drug toxicity or acute interstitial nephritis, vascular thrombosis, or underlying glomerular disease. These mechanisms often coexist or evolve, so edema and creatinine alone cannot direct therapy. This focused narrative review links the nephrotic substrate and precipitating exposures to predominant or mixed AKI phenotypes, multimodal assessment, and phenotype-guided management. Serum creatinine, urine output, and cystatin C are considered alongside urinary neutrophil gelatinase-associated lipocalin (NGAL), kidney injury molecule-1 (KIM-1), and the cellular stress product of tissue inhibitor of metalloproteinases-2 and insulin-like growth factor-binding protein 7 [TIMP-2]·[IGFBP7]. These biomarkers may help distinguish functional filtration loss from tubular stress or damage, but nephrotic-syndrome-specific validation and evidence of therapeutic utility remain limited. Management should integrate serial assessment of perfusion, congestion, fluid balance, urine findings, drug exposures, infection, and vascular or intrinsic kidney disease. Biomarkers are therefore adjuncts, not an established basis for management.

## 1. Introduction

Childhood nephrotic syndrome is characterized by nephrotic-range proteinuria and hypoalbuminemia, usually with edema. Although most cases are steroid sensitive, relapses, steroid resistance, infection, thrombosis, treatment toxicity, and acute kidney injury (AKI) can complicate the course [1]. Among hospitalized children with nephrotic syndrome, a meta-analysis estimated an AKI incidence of 29%, despite substantial variation in populations, baseline-creatinine assumptions, and definitions [2]. A multicenter cohort found AKI in more than half of hospitalized children, with longer hospitalization and greater intensive-care use [3]. These findings establish the clinical burden but not the mechanism in an individual child.

Serum creatinine and urine output identify functional deterioration but not the underlying lesion or pathophysiological mechanism [4]. In nephrotic syndrome, hypoalbuminemia, altered Starling forces, primary renal sodium retention, edema, infection, and drug exposures may act together. Some episodes fit the underfill model, in which reduced plasma oncotic pressure promotes interstitial fluid shift, low effective arterial volume, and secondary sodium retention. Others involve primary sodium retention and extracellular expansion, but edema cannot distinguish these states [5,6]. Thus, similar presentations may require opposite interventions: cautious restoration of effective circulating volume or controlled decongestion. Misclassification may add a preventable second insult.

The differential diagnosis also includes tubular injury from prolonged hypoperfusion, sepsis, protein overload, or nephrotoxins, as well as drug-induced acute interstitial nephritis (AIN), calcineurin-inhibitor toxicity, renal venous thrombosis, and active glomerular disease [7,8]. Because mechanisms may overlap or evolve, a label assigned at presentation can become inaccurate after fluids, diuresis, infection progression, or continued nephrotoxic exposure.

**Figure 1:**
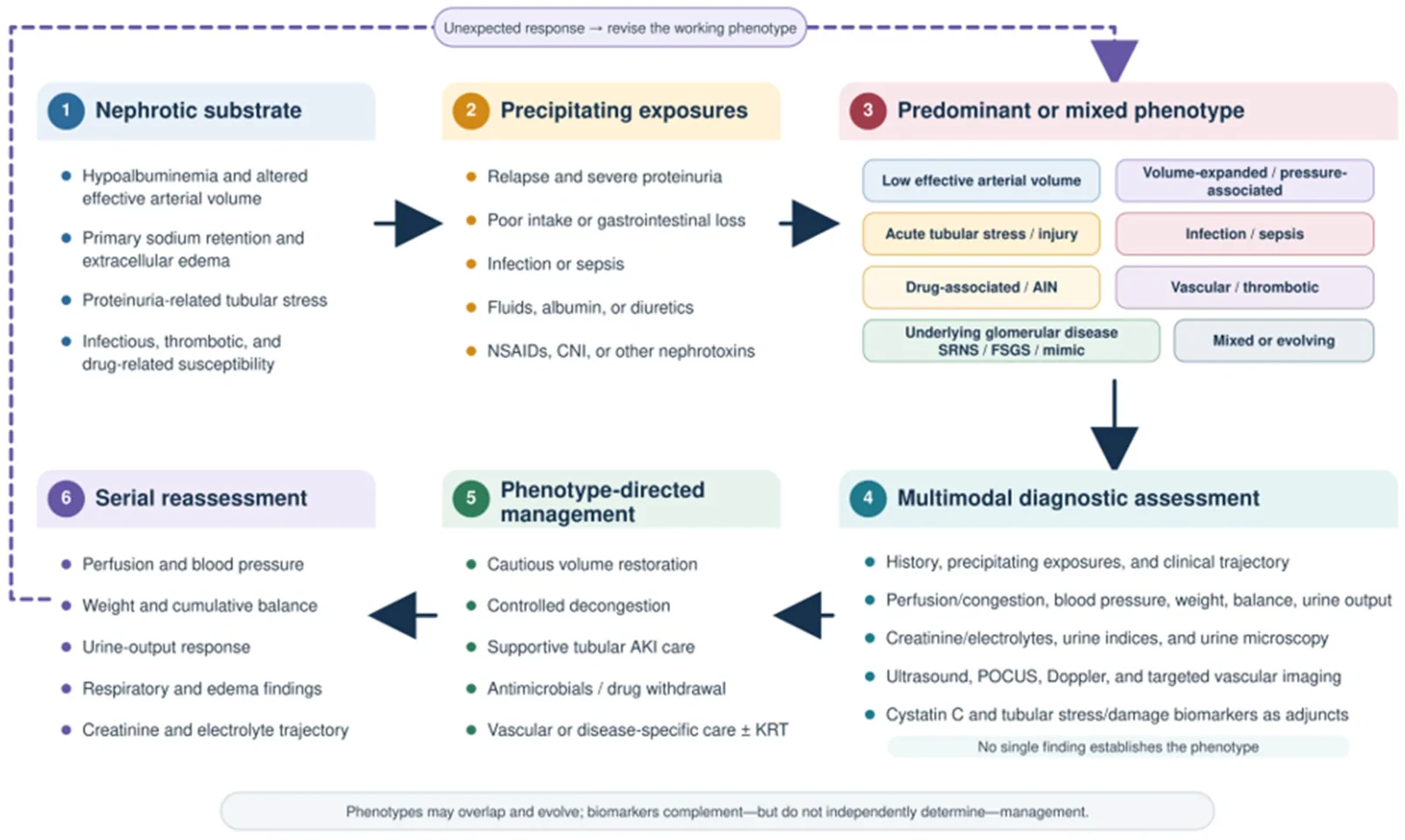
Mechanism-based framework for AKI in childhood nephrotic syndrome. Nephrotic disease and acute exposures may produce distinct or overlapping AKI phenotypes. Assessment combines the clinical course, perfusion and congestion, blood pressure, weight, fluid balance, urine output, laboratory and urinary findings, imaging, POCUS, and, when available, functional and tubular biomarkers. Management targets the working phenotype and requires serial reassessment. An absent or unexpected response should prompt reconsideration because mechanisms may coexist or evolve. Biomarkers are complementary and should not determine treatment alone. AIN, acute interstitial nephritis; AKI, acute kidney injury; CNI, calcineurin inhibitor; FSGS, focal segmental glomerulosclerosis; KRT, kidney replacement therapy; NSAIDs, nonsteroidal anti-inflammatory drugs; POCUS, point-of-care ultrasonography; SRNS, steroid-resistant nephrotic syndrome.

Emerging biomarkers may bridge functional deterioration and tissue-level stress or damage. Cystatin C complements creatinine as a filtration marker, whereas neutrophil gelatinase-associated lipocalin (NGAL), kidney injury molecule-1 (KIM-1), and the product of tissue inhibitor of metalloproteinases-2 and insulin-like growth factor-binding protein 7, [TIMP-2]·[IGFBP7], provide tubular damage or cellular stress signals. The Acute Disease Quality Initiative (ADQI) framework combines functional and damage biomarkers, but positive results are not cause-specific and may be influenced by proteinuria, inflammation, age, corticosteroids, and urine concentration [9]. This review links mechanism, phenotype, bedside discrimination, management, and recovery (Figure 1), treating AKI as a dynamic multimodal problem in which biomarkers complement rather than determine clinical decisions.

## 2. Materials and Methods

This focused narrative review was informed by an iterative PubMed search from database inception through 31 July 2026. Core search terms included “nephrotic syndrome,” “childhood” or “pediatric,” and “acute kidney injury.” Additional searches addressed volume-related mechanisms, including hypovolemia, effective arterial volume, edema, sodium retention, congestion, and renal interstitial pressure. Other terms covered tubular injury, infection, sepsis, nephrotoxicity, AIN, thrombosis, and steroid-resistant nephrotic syndrome. Diagnostic and outcome terms included ultrasonography, point-of-care ultrasonography (POCUS), cystatin C, NGAL, KIM-1, TIMP-2, IGFBP7, acute kidney disease, recovery, and follow-up. Reference lists of key guidelines, consensus reports, systematic reviews, and directly relevant studies were screened to identify additional publications. Evidence selection was purposefully focused rather than exhaustive. Priority was given to pediatric nephrotic-syndrome cohorts, prospective studies, systematic reviews, and contemporary guidelines, while older studies were retained when they provided foundational physiological or histopathological evidence. When direct pediatric evidence was absent, evidence from general pediatric AKI, adult minimal-change disease, critical care, or experimental models was used explicitly as indirect or mechanistic support. Study design, population, AKI definition, directness, and consistency were considered during interpretation. No formal risk-of-bias instrument, quantitative synthesis, or Preferred Reporting Items for Systematic Reviews and Meta-Analyses (PRISMA) flow diagram was applied because the objective was a focused narrative synthesis. Conclusions were calibrated to the strength and applicability of the available evidence, and proposed phenotype and biomarker frameworks were treated as conceptual rather than validated clinical algorithms.

## 3. Clinical Burden and Limits of Creatinine-Based AKI Classification

### 3.1. Definition and Staging

Kidney Disease: Improving Global Outcomes (KDIGO) defines AKI as a serum-creatinine increase of at least 0.3 mg/dL within 48 h, an increase to at least 1.5 times baseline within seven days, or urine output below 0.5 mL/kg/h for six hours. Stages 1–3 reflect creatinine change, oliguria, and kidney replacement therapy [10], and pediatric stage 3 also includes estimated glomerular filtration rate (eGFR) below 35 mL/min/1.73 m^2^. In nephrotic syndrome, unknown or very low baseline creatinine, dilution by fluid accumulation, and non-steady-state kinetics limit interpretation and invalidate routine eGFR assumptions [1,4].

### 3.2. Incidence, Risk Factors, and Short-Term Outcomes

Reported AKI frequency varies with case mix and definition. A United States hospitalization analysis found a 158% increase in nephrotic-syndrome admissions complicated by AKI from 2000 to 2009 [11]. A subsequent multicenter cohort identified AKI in 58.6% of 336 children and 50.9% of 615 hospitalizations [3], whereas a national childhood-onset cohort reported AKI in approximately one third of hospitalizations [12]. Other studies differed in relapse status, steroid response, referral severity, and pediatric Risk, Injury, Failure, Loss, and End-stage kidney disease (pRIFLE) or KDIGO criteria [13,14]. Prospective evidence remains single-center and hospitalization based [15]. Across 11 studies, the pooled incidence was 29% (95% confidence interval, 23–37%), with substantial heterogeneity [2].

AKI clustered with infection, hypertension, lower serum albumin, steroid-resistant or more severe disease, and nephrotoxic or hemodynamically active drugs [3,14,15]. Additional associations included age at nephrotic-syndrome onset, sex, steroid response, and medication exposure, but observational data do not establish independent causal mechanisms [2]. In a multicenter cohort, each additional nephrotoxic medication increased AKI risk, and affected children required longer hospitalization and more intensive care [3]. Recovery is common, although greater severity predicts slower or incomplete early recovery [13]. AKI therefore identifies a complex hospitalization, and its observed frequency depends partly on admission practices and disease severity.

### 3.3. Why the Creatinine Phenotype Is Mechanistically Non-Specific

Creatinine is a delayed index of functional change influenced by production, distribution volume, and tubular handling, while urine output is more immediate but varies with sodium-retentive nephrosis, diuretics, fluid intake, and collection accuracy [4]. The same KDIGO stage may therefore reflect underperfusion, pressure-associated filtration impairment, tubular injury, infection or sepsis, drug-related hemodynamic change, toxicity, AIN, thrombosis, or glomerular disease [7,8]. The ADQI framework recognizes that functional deterioration may occur with or without a damage signal and that tubular stress may precede conventional AKI [9]. Creatinine staging remains essential for severity and prognosis but should initiate, not conclude, mechanistic assessment.

## 4. Why Children with Nephrotic Syndrome Are Vulnerable to AKI

### 4.1. Underfill Physiology and Reduced Effective Circulating Volume

In the underfill model, urinary albumin loss lowers plasma oncotic pressure and shifts fluid into the interstitium. When lymphatic and vascular compensation fails, effective circulating volume decreases, activating the renin-angiotensin-aldosterone system, sympathetic activity, and vasopressin. The resulting sodium and water retention may preserve perfusion but worsen edema [16,17]. This phenotype is most plausible with abrupt severe hypoalbuminemia and rapidly evolving edema, especially with poor intake, vomiting, diarrhea, fever, fluid restriction, or intensive diuresis [5,6]. Its expression depends on the rate of albumin loss, interstitial compliance, lymphatic adaptation, and superimposed fluid losses rather than a single albumin threshold. NSAIDs or renin-angiotensin system blockade may disrupt autoregulation and expose a functional glomerular filtration rate (GFR) decline that can progress to tubular injury. Hypoalbuminemia indicates susceptibility, not circulating volume, and generalized edema does not exclude intravascular depletion.

### 4.2. Overfill and Primary Renal Sodium Retention

The overfill model begins with primary renal sodium retention rather than compensation for arterial underfilling. During active nephrosis, sodium excretion may remain low despite suppressed or inactive renin-aldosterone pathways [5,17]. Filtered serine proteases can activate epithelial sodium channels in the distal nephron. Plasmin contributes, but redundant proteases and other tubular pathways prevent attribution to a single enzyme [18]. Reduced natriuretic responsiveness and altered intrarenal hemodynamics may add to sodium retention.

Extracellular expansion may present with hypertension, rapid weight gain, tense edema, ascites, or respiratory compromise. Pediatric bioimpedance studies document increased extracellular water during active disease, although hormonal, natriuretic-peptide, and sonographic measures are not consistently concordant [6]. In a prospective study, selected volume-expanded children achieved edema reduction with diuretics alone, showing that albumin is not universally required [19]. Expansion does not by itself prove pressure-mediated AKI, and urine sodium may remain low. Underfill and overfill are working physiological patterns that may coexist.

### 4.3. Renal Interstitial Edema, Venous/Tissue Pressure, and “Renal Tamponade”

Because the kidney is enclosed by a relatively noncompliant capsule, severe interstitial edema may increase intrarenal pressure, compress tubules and capillaries, and reduce filtration. Renal venous hypertension or increased intra-abdominal pressure may worsen these effects [20,21]. A similar “renal tamponade” mechanism has been proposed in severe minimal-change nephrotic syndrome [8], but supporting evidence is largely derived from adult studies and histopathological or mechanistic observations. No validated pediatric criteria define a pressure-associated phenotype, so edema alone cannot confirm this mechanism.

### 4.4. Proteinuria-Induced Tubular Stress

Massive protein traffic is biologically active. Proximal tubular uptake of albumin and other filtered proteins can trigger oxidative, inflammatory, profibrotic, complement, and chemokine pathways, while luminal protein and debris may promote casts and obstruction [22,23]. Increased reabsorptive oxygen demand may further reduce reserve during hypoperfusion. The resulting continuum from reversible stress to structural injury is most relevant with severe or prolonged proteinuria and superimposed ischemia, infection, or nephrotoxicity. Experimental and mixed-age nephrotic evidence links protein loading to KIM-1 and NGAL expression and biopsy-detected tubular abnormalities [24], without showing additional short-term marker elevation from furosemide. These data support protein-overload stress but neither prove that proteinuria alone causes pediatric AKI nor allow urinary KIM-1 or NGAL to distinguish the nephrotic substrate from a new tubular insult.

### 4.5. Cross-Cutting Susceptibilities

Cross-cutting risks may occur with either volume pattern. Urinary losses of immunoglobulins and complement, edema, invasive devices, and immunosuppression increase susceptibility to infection. Major infections remain common among hospitalized children with nephrotic syndrome [25]. Fever, gastrointestinal fluid losses, vasodilation, capillary leak, and antimicrobials can then converge on AKI. NSAIDs, renin-angiotensin system blockers, calcineurin inhibitors, diuretics, and nephrotoxic antibiotics may be combined. Infection, steroid-resistant disease, and cumulative nephrotoxin exposure were associated with AKI in hospitalized cohorts [3]. Systematic exposure review and serial creatinine surveillance can reduce medication-associated pediatric AKI, although evidence is not nephrotic-syndrome specific [26].

Age should inform assessment alongside disease characteristics and treatment history. Congenital and infantile nephrotic syndrome require separate etiologic and management considerations from typical childhood steroid-sensitive disease, while adolescent-onset nephrosis warrants particular attention to atypical or secondary glomerular disease [1]. In children with established nephrotic syndrome, volume losses, infection, and medication exposure remain relevant across age groups [3,7]. Age therefore provides clinical context but does not independently establish the mechanism of an AKI episode.

Compared with adults, childhood idiopathic nephrotic syndrome is more commonly associated with minimal change disease, often presumed from a typical steroid-sensitive presentation. AKI may complicate volume depletion or excessive diuresis, infection, and nephrotoxic exposure [1,3,7]. Adult nephrotic syndrome encompasses a broader etiologic spectrum, while hypertension, diabetes, pre-existing kidney disease, and medication exposure may add to AKI susceptibility [8,21]. These differences concern the underlying disease and susceptibility rather than mutually exclusive mechanisms. In children, serial assessment of perfusion, fluid balance, and response to treatment is particularly important because edema alone cannot establish intravascular volume status [1,7].

## 5. Predominant Mechanistic Phenotypes

### 5.1. Low Effective Arterial Volume/Hemodynamic Phenotype

The low-effective-arterial-volume phenotype is a predominantly functional GFR reduction caused by inadequate renal perfusion. Rapid interstitial sequestration may coexist with low venous return during profound hypoalbuminemia, poor intake, gastrointestinal loss, fever, or intensive diuresis. Sustained hypoperfusion, NSAID-mediated loss of afferent vasodilation, or renin-angiotensin system blockade can raise creatinine and eventually injure tubules [5,7]. Pediatric studies do not support universal underfilling because estimates varied when biochemical indices and inferior vena cava (IVC) collapsibility were used [27,28,29], and a lower IVC-to-aorta ratio during relapse did not independently identify clinically defined low intravascular volume [30]. Because these studies used non-equivalent volume-status standards and did not address AKI causation, diagnosis requires convergent chronology, examination, laboratory findings, and monitored response to cautious volume restoration.

### 5.2. Volume-Expanded/Congestion- or Pressure-Associated Phenotype

The volume-expanded phenotype reflects primary sodium retention and may involve epithelial sodium channel activation by filtered proteases [18]. Small pediatric bioimpedance studies documented increased extracellular water during active nephrosis, but bioimpedance, N-terminal pro-B-type natriuretic peptide (NT-proBNP), IVC, and hormonal measures showed limited concordance [31,32]. Selected volume-expanded children lost edema with diuretics alone, supporting a volume-expanded clinical pattern, although a causal link between expansion and pediatric AKI remains unproven [19]. Potential pressure mechanisms include renal venous hypertension, raised intra-abdominal pressure, and interstitial compression of tubules or peritubular capillaries [20,21]. The label therefore denotes a plausible, not validated, pediatric phenotype.

### 5.3. Acute Tubular Stress/Injury Phenotype

Acute tubular stress or injury may follow prolonged hypoperfusion, sepsis, nephrotoxins, or heavy protein traffic. Filtered proteins activate oxidative, inflammatory, and profibrotic pathways, while casts and interstitial edema may obstruct tubular flow [22,23]. Adult minimal-change disease can show acute tubular injury or necrosis on biopsy, but pediatric histological evidence is limited and concurrent insults impede attribution [7,8]. Experimental and mixed-age evidence links protein exposure to higher KIM-1 and NGAL expression and tubular abnormalities, indicating that proteinuria itself can generate a stress signal [24]. A pediatric single-center study likewise found higher urinary KIM-1 and NGAL in established AKI and in higher KDIGO stages but did not demonstrate pre-AKI prediction or distinguish protein-overload stress from structural injury [33]. Persistent oliguria, granular casts, biomarker trajectories, and nonresponse after hemodynamic correction increase suspicion but are not independently diagnostic.

### 5.4. Infection-, Sepsis-, Drug-, and Tubulointerstitial Inflammation-Associated Phenotypes

Infection-associated AKI may combine impaired intake, extrarenal fluid losses, vasodilation, capillary leak, microcirculatory disturbance, inflammation, and antimicrobial exposure. Sepsis-associated AKI is not simply “prerenal” because endothelial dysfunction, dysregulated immunity, and metabolic adaptation can produce tubular stress despite variable global renal blood flow [34]. Infection and nephrotoxins were independently associated with AKI in hospitalized children with nephrotic syndrome, without defining the mechanism in an individual patient [3]. A single-center prospective study confirmed the burden of septic AKI in steroid-responsive nephrotic syndrome but did not provide mechanistic phenotyping [35]. Drug-associated AKI may reflect hemodynamic effects from diuretics or renin-angiotensin system inhibitors, NSAID-related afferent vasoconstriction, calcineurin-inhibitor vasoconstriction, or aminoglycoside tubular toxicity [7]. Antibiotics, NSAIDs, and other agents may instead cause AIN, often without fever, rash, and eosinophilia [36,37]. Because no noninvasive finding confirms drug-induced AIN, exposure chronology, urinalysis, systemic findings, competing diagnoses, and sometimes biopsy must be integrated. These processes may coexist but remain diagnostically distinct.

### 5.5. Vascular and Thrombotic Phenotype

Nephrotic hypercoagulability reflects urinary loss of anticoagulant proteins, increased procoagulant factors, platelet activation, and hemoconcentration, compounded by immobility and venous catheters [38,39]. Thromboembolism affects 3.60% of children with nephrotic syndrome, rising in congenital and steroid-resistant disease [40]. Older age, severe proteinuria, and previous thrombosis were independent predictors in the Midwest consortium cohort, and nearly half the events were catheter-associated [41]. AKI may occur with bilateral or extensive renal venous thrombosis, whereas unilateral disease typically produces flank pain, hematuria, or renal enlargement. Infection and venous thromboembolism co-occurred in children, although causality was not established [42]. Unexplained dysfunction with symptoms or imaging asymmetry should prompt vascular evaluation rather than attribution to hemodynamic AKI.

### 5.6. Underlying Glomerular Disease, SRNS/FSGS, and Mimics

Underlying glomerular disease is a clinical context rather than a single AKI mechanism. Active minimal-change disease may modestly and reversibly reduce GFR through altered ultrafiltration without necessarily causing tubular necrosis [8]. In steroid-resistant nephrotic syndrome or focal segmental glomerulosclerosis (FSGS), proteinuria, scarring, hypertension, reduced reserve, and calcineurin-inhibitor exposure increase vulnerability. Rising creatinine may instead indicate chronic kidney disease (CKD) progression [43]. Severe AKI occurred in 25.8% of children in a non-genetic steroid-resistant nephrotic syndrome cohort, often in association with infection or posterior reversible encephalopathy syndrome, although follow-up kidney outcomes were favorable [44]. In children with secondary steroid resistance, AKI was associated with subsequent kidney failure, although the retrospective design and disease severity limit inference [45]. Pyelonephritis, thrombosis, AIN, tubular injury, and progressive glomerular disease remain alternative causes of kidney dysfunction [46]. Persistent dysfunction or an atypical urinary sediment warrants further evaluation, including kidney biopsy, genetic testing, or both, when indicated.

### 5.7. Mixed and Evolving Phenotypes

Phenotypes may coexist and evolve over time, with hemodynamic dysfunction progressing to tubular injury, sepsis involving vasodilation, capillary leak, inflammation, and nephrotoxin exposure, and infection coinciding with thrombosis [7,34,42]. Edema or extracellular expansion does not exclude low effective arterial volume. Assessment should identify the predominant mechanism at a defined time, record secondary contributors, and be repeated after intervention. An unexpected creatinine or urine-output response should prompt reclassification rather than automatic intensification of the initial therapy. Table 1 summarizes the mechanisms, contexts, and diagnostic limitations.

## 6. Diagnostic Phenotyping at the Bedside

### 6.1. Clinical Timeline and Examination

Phenotyping begins with a review of the clinical course, including nephrotic activity, weight changes, oral intake, gastrointestinal losses or fever-related fluid loss, sodium and fluid intake, albumin and diuretic administration, exposure to nephrotoxic or hemodynamically active drugs, symptoms of infection, and the onset of oliguria and creatinine elevation. Examination should assess both impaired perfusion and extracellular expansion rather than treat edema as a volume diagnosis. Heart rate, capillary refill, extremity temperature, mental state, orthostatic symptoms, and blood pressure provide context, although hypotension is late in children. Hypertension, tense edema or ascites, respiratory distress, crackles, or rising oxygen need may support expansion or pressure-related dysfunction [5,6]. Serial weight, urine output, fluid balance, perfusion, and respiratory findings are more informative than a single examination, because edema and low effective arterial volume may coexist.

### 6.2. Laboratory and Urinary Assessment

Serum creatinine should be interpreted against the most credible pre-illness baseline and followed as a trajectory with urine output, urea, electrolytes, bicarbonate, albumin, and hematological findings. Cultures, inflammatory tests, hemolysis indices, complement, or serology should be targeted to the suspected infectious, thrombotic, microangiopathic, or glomerular process. Urinalysis and microscopy can materially redirect the working phenotype: a bland sediment may fit functional hemodynamic change, granular casts or tubular epithelial cells support tubular injury, pyuria may suggest infection or AIN, and dysmorphic erythrocytes or red-cell casts require evaluation for active glomerular disease. Absence of these findings does not exclude the corresponding lesion [7].

Urine sodium and fractional excretion indices are adjuncts, not binary tests. Low urine sodium or fractional excretion of sodium may reflect either true underfilling or nephrotic sodium retention. Diuretics, sepsis, CKD, timing, and recent fluid or albumin administration further limit interpretation, and fractional excretion of urea has similar constraints. Serum albumin also cannot define intravascular volume. These results require concordance with chronology, examination, sediment, and subsequent course [27,28].

### 6.3. Ultrasound, Doppler, and POCUS

Kidney and bladder ultrasonography is useful for excluding obstruction and assessing kidney size, echogenicity, or asymmetry, but uncomplicated hemodynamic and tubular AKI usually lack specific grayscale findings. Doppler evaluation becomes urgent when renal venous or arterial thrombosis is suspected, and a negative or technically limited study should not terminate evaluation when clinical probability remains high. POCUS can provide information on cardiac function, lung B-lines or pleural fluid, IVC behavior, and, in experienced hands, venous Doppler patterns. These measurements should be interpreted as components of a physiological profile rather than direct measures of effective arterial volume.

Pediatric nephrotic studies used non-equivalent definitions and reference standards, limiting interpretation of IVC indices [27,28,29]. A lower IVC-to-aorta ratio during relapses did not independently identify low intravascular volume [30], and bioimpedance, NT-proBNP, and IVC sonography showed incomplete concordance [31,32]. POCUS is therefore an adjunct for serial multimodal assessment, not a validated stand-alone discriminator.

Venous excess ultrasound (VExUS) grading, originally developed in adults, combines IVC assessment with hepatic, portal, and intrarenal venous Doppler to grade systemic venous congestion from 0 to 3 [48]. Preliminary pediatric experience includes a four-patient conference report involving one child with nephrotic syndrome [49]. However, pediatric nephrotic-syndrome-specific grading criteria and treatment thresholds remain unvalidated, and the potential role of VExUS as an adjunct for serial congestion assessment requires prospective study.

### 6.4. An Integrated and Dynamic Working Phenotype

Bedside assessment should yield a provisional statement of the predominant mechanism, secondary contributors, severity, and main alternative diagnosis. Table 1 organizes supportive and contradictory clues. Response to a cautious intervention, assessed through perfusion, blood pressure, weight, respiratory findings, urine output, and creatinine, adds evidence but is not an infallible diagnostic test. An unexpected response should trigger review of chronology, drugs, sediment, imaging, and mixed or evolving injury. No single history item, fractional excretion index, ultrasound measure, or biomarker can replace serial synthesis.

## 7. Conventional and Emerging Biomarkers

### 7.1. Functional Biomarkers: Serum Creatinine, Urine Output, and Cystatin C

Serum creatinine and urine output remain the KDIGO anchors for AKI diagnosis and staging, but they measure functional consequences rather than tissue injury [10]. Creatinine rises after GFR has fallen, is diluted by fluid accumulation, and varies with muscle mass and tubular secretion [4,50]. Urine output may signal deterioration earlier, although incomplete collection, diuretics, and nephrotic sodium retention complicate interpretation. Cystatin C is less muscle-dependent and may identify functional change earlier, but corticosteroids and thyroid dysfunction are relevant non-GFR determinants [50]. A 2026 pediatric meta-analysis reported pooled sensitivity of 78.2%, specificity of 79.5%, and an area under the curve of 0.854, with variation by setting and threshold [51]. Its reference standards were conventional AKI definitions, however, and it was not nephrotic-syndrome specific. A small childhood nephrotic-syndrome study found associations of cystatin C and NGAL with kidney function and disease variables but did not evaluate diagnostic accuracy for AKI [52].

### 7.2. Tubular Damage Biomarkers: NGAL and KIM-1

Urinary NGAL and KIM-1 provide signals distinct from filtration. NGAL increases after tubular stress but may also originate from activated neutrophils and extrarenal inflammation, whereas KIM-1 is expressed by injured proximal tubular epithelial cells. Across heterogeneous pediatric settings, urinary NGAL shows useful overall discrimination for AKI, but performance varies by population, sampling, assay, and reference standard [53,54]. Evidence for KIM-1 is less consistent, with a pediatric meta-analysis reporting an overall area under the curve of 0.69, with better early discrimination when sampled within 12 h [55].

Direct childhood nephrotic-syndrome evidence is limited. A single-center study found higher urinary NGAL and KIM-1 in 47 children with established AKI than in 169 without AKI, with higher values in KDIGO stages 2–3 than stage 1 [33]. Because conventional criteria defined groups and sampling did not establish pre-creatinine prediction, the findings indicate association with established AKI and severity, not mechanistic discrimination. Proteinuria itself may generate tubular signals: mixed-age mechanistic evidence linked protein loading to cellular NGAL and KIM-1 expression and frequent biopsy abnormalities [24]. In steroid-dependent or steroid-resistant disease, elevated markers may also reflect persistent proteinuria and chronic tubulointerstitial involvement [56]. Neither marker alone can therefore distinguish hemodynamic dysfunction from structural injury.

### 7.3. Cellular Stress Biomarkers: [TIMP-2]·[IGFBP7]

Urinary [TIMP-2]·[IGFBP7] reflects cell-cycle arrest associated with tubular stress and may precede functional loss. In a heterogeneous cohort containing 46 established AKI cases, higher values were associated with severe AKI and adverse outcomes, supporting prognostic rather than pre-AKI diagnostic use [57]. Among children receiving cisplatin, discrimination was poor to modest and varied with sampling and urinary-creatinine indexing (areas under the curve approximately 0.61–0.70) [58]. A pediatric meta-analysis reported pooled sensitivity of 0.84, specificity of 0.85, and an area under the curve of 0.91, but performance differed by setting and platform, with possible publication bias [59]. No childhood nephrotic-syndrome validation exists, so this remains a research-level stress signal, not a treatment-determining threshold.

### 7.4. Nephrotic-Syndrome-Specific Interpretation Problems

Nephrotic disease itself shapes biomarker concentrations. Proteinuria can provoke tubular uptake, inflammation, NGAL/KIM-1 expression, and urinary-matrix changes [24]. Persistent proteinuria and tubulointerstitial remodeling may elevate baseline markers without a new insult [56], while disease phase and baseline kidney function may explain differences in small cohorts [52].

Nonrenal and analytical modifiers also matter. Infection and systemic inflammation can increase NGAL while representing genuine sepsis-associated tubular stress, thereby reducing etiologic specificity [34]. Corticosteroids and thyroid status affect cystatin C independently of GFR [50], and healthy pediatric concentrations of urinary NGAL and KIM-1 vary with age and sex [60]. Reference distributions derived from healthy children are not validated AKI diagnostic thresholds in nephrotic syndrome and should be interpreted within the age range and assay conditions in which they were established. Values from older children should not be extrapolated to neonates or infants. Urine flow, diuretics, sampling time, and creatinine indexing also alter concentrations [58]. Universal cutoffs are therefore inappropriate for isolated measurements. Interpretation should combine serial values, baseline or remission samples, assay-specific pediatric ranges, proteinuria, infection, nephrotoxins, volume phenotype, and functional change. Biomarkers may identify stress or damage, but not its cause. Without a pre-AKI or remission baseline, NGAL and KIM-1 may still provide supportive evidence of tubular stress, but an isolated elevation cannot establish a new injury superimposed on active nephrosis. No absolute or relative change between serial measurements has been validated for identifying superimposed AKI in pediatric nephrotic syndrome. A rising trajectory may increase concern when accompanied by worsening kidney function, reduced urine output, or supportive urinary sediment findings. However, serial comparisons require consistent assays and normalization methods and concurrent assessment of proteinuria, infection, and urine concentration. Table 2 summarizes their contribution, limitations, and readiness.

### 7.5. A Functional and Structural AKI Matrix and Clinical Applicability

Combining functional and stress or damage signals may add information [9]. Functional change includes creatinine, urine output, and potentially cystatin C, whereas stress or damage signals include NGAL, KIM-1, and [TIMP-2]·[IGFBP7]. Positivity does not prove structural or irreversible injury, and negativity does not exclude early or focal damage. Without nephrotic-syndrome-specific thresholds or trajectories, the matrix supports reassessment and research stratification but cannot independently determine albumin administration, diuresis, antimicrobial therapy, nephrotoxin withdrawal, imaging, or kidney replacement therapy. Clinical utility beyond conventional assessment requires prospective testing. A 2025 single-center study in critically ill children with established AKI found that combining individual urinary biomarkers—NGAL, TIMP-2, IGFBP7, or C–C motif chemokine ligand 14—with concurrent creatinine-based AKI staging improved prediction of persistent and prolonged AKI [61]. These findings support integrated prognostic assessment but require validation in childhood nephrotic syndrome. Table 3 presents the four possible marker combinations.

## 8. Phenotype-Guided Management

### 8.1. Universal Initial Measures

Initial care requires accurate urine output and fluid balance, serial weight and blood pressure, examination for impaired perfusion and pulmonary edema, and repeated creatinine, electrolyte, and acid-base assessment. Medication review should identify potential nephrotoxins and hemodynamically active drugs and ensure dose adjustment of renally eliminated agents, although kidney-function estimates are unreliable while creatinine is changing [10]. Nephrotic activity and immunosuppression must also be reassessed [1]. Structured nephrotoxin surveillance with daily creatinine reduced medication-associated AKI in high-risk hospitalized children, although evidence was not nephrotic-syndrome specific [26]. Early pediatric-nephrology involvement is appropriate for severe, persistent, or mechanistically uncertain AKI.

### 8.2. Low-Volume/Hemodynamic Phenotype

When integrated assessment supports reduced effective arterial volume, diuretics and renin-angiotensin system blockers should initially be withheld and volume restored cautiously. Isotonic crystalloid is appropriate for shock or documented extrarenal fluid loss. Concentrated albumin may be considered for severe edema with convincing intravascular depletion, but hypoalbuminemia or edema alone is not an indication [1,62]. Evidence for albumin plus furosemide in childhood nephrotic edema is limited, heterogeneous, and focused on short-term physiological outcomes [63]. The development of hypertension, pulmonary edema, or worsening oliguria during administration requires immediate reassessment.

### 8.3. Management of the Volume-Expanded/Congestive Phenotype

Clinically volume-expanded children require controlled decongestion without reflex crystalloid or albumin. Sodium restriction is appropriate for moderate-to-severe edema, whereas fluid restriction should be individualized rather than routine [62]. Loop diuretics are first-line therapy, with intravenous administration when intestinal edema limits absorption. Before sequential nephron blockade, apparent resistance should prompt review of dose, delivery, adherence, kidney function, and phenotype. A small prospective study reported successful diuresis without albumin in selected children with severe edema (≥3+ pitting edema and ascites), classified as volume expanded using fractional excretion of sodium ≥0.2% in its second phase [19]. The exclusion of children with reduced GFR or rising serum creatinine limits extrapolation to established AKI. Refractory fluid overload with organ compromise warrants kidney replacement therapy (KRT) evaluation.

### 8.4. Acute Tubular Stress/Injury

Supportive management includes prevention of further nephrotoxic exposure, maintenance of adequate but not excessive perfusion, correction of electrolyte and acid-base disturbances, and adjustment of drug doses. Diuretics may control overload but do not treat tubular injury or accelerate recovery, and forced diuresis is inappropriate. Mixed-age evidence showed no additional short-term increase in urinary KIM-1 or NGAL with furosemide but does not establish safety in pediatric AKI or override phenotype-based prescribing [24].

### 8.5. Infection, Sepsis, and Tubulointerstitial Injury

Suspected sepsis requires cultures when feasible, prompt empiric antimicrobial therapy, and source control without delaying treatment in an unstable child, and selection, dosing, and de-escalation should follow kidney function and microbiology results [64]. Hemodynamic support must remain phenotype-sensitive because infection can coexist with depletion, capillary leak, or fluid accumulation, requiring reassessment after each intervention [34]. For suspected AIN, withdraw the likely causative drug [36]. Kidney biopsy is reserved for severe, uncertain, or non-resolving cases. Evidence for corticosteroids in drug-induced AIN is observational, so treatment should be individualized after excluding active infection and obtaining specialist assessment [37].

### 8.6. Drug-Associated AKI

Drug-associated AKI should be classified mechanistically: hemodynamic GFR reduction, direct tubular toxicity, AIN, calcineurin-inhibitor vasoconstriction, or endothelial injury/thrombotic microangiopathy. NSAID exposure is associated with increased AKI risk in hospitalized children [65]. Suspected agents should be stopped, reduced, or replaced whenever possible. For calcineurin inhibitors, trough concentration, interacting drugs, blood pressure, potassium, and evidence of hemolysis or thrombocytopenia should be assessed, although therapeutic-range concentrations do not exclude nephrotoxicity [47]. Reintroduction of antiproteinuric or immunosuppressive therapy should follow renal recovery and reassessment of benefits and risks.

### 8.7. Vascular/Thrombotic Phenotype

Flank pain, gross hematuria, asymmetric edema, catheter dysfunction, unexplained thrombocytopenia, or abrupt AKI should raise suspicion of renal vascular disease. Urgent Doppler or other site-directed imaging should match the suspected site and clinical stability. Confirmed thrombosis requires multidisciplinary, event-specific anticoagulation or intervention, but nephrotic syndrome alone does not justify indiscriminate prophylaxis because thrombotic and bleeding risks vary [39]. The pediatric burden supports vigilance in high-risk disease without justifying routine prophylaxis [40].

### 8.8. Severe or Refractory AKI

KRT is indicated for complications refractory to medical treatment, including fluid overload with organ compromise, dangerous hyperkalemia, severe metabolic acidosis, or uremic manifestations, whereas serum creatinine alone is not an indication [10]. Modality selection depends on hemodynamic status, illness severity, local expertise, and resources. Peritoneal dialysis is an accepted modality for pediatric AKI [66]. Greater fluid overload at continuous KRT initiation is associated with higher mortality in critically ill children, but this observational evidence is not nephrotic-syndrome-specific and does not establish a universal percentage threshold for starting KRT [67].

### 8.9. Dynamic Reassessment

The assigned phenotype remains provisional. Reassess perfusion, blood pressure, weight and fluid balance, urine output, respiratory findings, and creatinine trajectory. An unexpected response, new hypertension or pulmonary edema, persistent oliguria, or systemic features should prompt reconsideration of diagnosis, drugs, imaging, and possible biopsy. Hemodynamic dysfunction may progress to tubular injury, and infection, nephrotoxicity, and congestion may coexist, so treatment should adapt as the phenotype evolves. Table 4 summarizes initial management, response measures, pitfalls, and escalation.

## 9. Recovery, Acute Kidney Disease, and Long-Term Follow-Up

Creatinine normalization does not complete AKI assessment. Recovery should be compared with pre-AKI function and classified as complete, partial, or absent. Persistent dysfunction during days 7–90 is acute kidney disease (AKD), whereas abnormalities beyond 90 days meet the temporal criterion for CKD [68]. Baseline ascertainment and pediatric recovery definitions nevertheless remain inconsistent.

Nephrotic-syndrome recovery is variable. In 68 AKI episodes, 88.2% recovered within three months, leukocyturia predicted slower recovery, and 11.9% developed recurrent AKI [69]. Recovery time increased with pRIFLE severity in another cohort [13]. Persistent three-month dysfunction was frequent in a prospective cohort enriched for steroid-resistant nephrotic syndrome, FSGS, and drug toxicity, limiting application to uncomplicated steroid-sensitive disease [70]. Primary steroid-resistant nephrotic syndrome showed no CKD during follow-up, whereas AKI in secondary steroid-resistant nephrotic syndrome was associated with kidney failure [44,45]. These heterogeneous populations cannot yield one causal estimate of post-AKI CKD risk.

General evidence supports surveillance. A large, matched cohort associated AKI with CKD, hypertension, KRT, and recurrent AKI over a median 9.7 years [71]. A recent meta-analysis reported pooled incidences of CKD, proteinuria, and hypertension of 17%, 20%, and 16%, respectively, although populations varied [72]. Post-discharge assessment should include creatinine/eGFR, blood pressure, and proteinuria interpreted against nephrotic activity. Formal reassessment is appropriate by three months [10], with earlier and longer nephrology follow-up after incomplete recovery, severe or recurrent AKI, KRT, persistent abnormalities, steroid-resistant nephrotic syndrome/FSGS, or continued nephrotoxic exposure.

## 10. Knowledge Gaps and Future Directions

The focused, non-exhaustive literature search and purposeful evidence selection in this narrative review may have introduced selection bias. The proposed phenotype-based clinical pathways and biomarker framework remain conceptual and require prospective validation before broader implementation in clinical practice. Major gaps extend from epidemiology to treatment. Different AKI definitions, baseline-creatinine assumptions, and hospitalized case mixes produce heterogeneous incidence estimates [2]. No validated pediatric reference standard distinguishes underfilling from volume-expanded or pressure-associated states. Available biochemical, IVC, and bioimpedance studies are small, single-center, and based on inconsistent surrogates [28,32], and the IVC-to-aorta index also failed to independently identify low intravascular volume [30]. Most studies evaluated edema or relapse rather than phenotype-defined AKI. POCUS and other volume measures therefore remain adjuncts pending external validation.

Biomarker evidence is less mature. One pediatric nephrotic-syndrome study linked higher urinary NGAL and KIM-1 to AKI and greater severity but did not establish mechanistic discrimination, validated thresholds, or treatment utility [33]. Proteinuria itself may increase tubular markers, while age, disease activity, infection, corticosteroids, and chronic tubulointerstitial injury modify concentrations [24]. Urinary [TIMP-2]·[IGFBP7] lacks nephrotic-syndrome-specific validation because pediatric evidence comes from heterogeneous neonatal, critical-care, surgical, and oncology settings with variable timing and platforms [58,59]. Age- and disease-specific thresholds require derivation and external validation.

Future multicenter cohorts should prespecify AKI, AKD, recovery, and phenotype definitions, document baseline-creatinine assumptions, and collect serial examinations, microscopy, POCUS, and biomarker data. Trajectories, incremental discrimination, and clinical utility should be tested beyond conventional assessment. No prospective pediatric nephrotic-syndrome trial has evaluated a prespecified phenotype- or biomarker-guided algorithm. Trials should compare cautious volume restoration with decongestion in well-characterized phenotypes and assess adverse events, hospitalization, KRT, recovery, AKD, and CKD. Structural biomarkers may complement functional criteria [9] but remain investigational adjuncts.

## 11. Conclusions

AKI in childhood nephrotic syndrome is not a unitary entity. Creatinine and urine output identify functional deterioration but not whether the predominant mechanism is hemodynamic, tubular, infectious, drug-related, vascular, or glomerular. Edema likewise does not define intravascular volume.

Management requires dynamic integration of clinical course, perfusion and congestion, blood pressure, serial weight, urine output, fluid balance, laboratory and urinary findings, and selected imaging. Because phenotypes overlap and evolve, classification remains provisional and must be reconsidered when response differs from expectation. Similar presentations may require cautious volume restoration or controlled decongestion.

Cystatin C, NGAL, KIM-1, and [TIMP-2]·[IGFBP7] may help distinguish functional GFR reduction from tubular stress or injury. However, nephrotic-syndrome-specific validation, standardized thresholds, and evidence of improved outcomes with biomarker-guided decisions are lacking. These markers should complement rather than independently determine therapy, as biomarker-guided management remains a goal rather than established practice.

## Figures and Tables

**Table 1:** Predominant Mechanistic Phenotypes of AKI in Childhood Nephrotic Syndrome.

Phenotype	Core Mechanism	Typical Context and Clinical Clues	Diagnostic Support and Limitations
Low effective arterial volume/hemodynamic	Reduced effective arterial volume and renal perfusion; prolonged dysfunction may cause tubular injury.	Fluid losses, poor intake, sepsis, excessive diuresis, or severe hypoalbuminemia. Tachycardia, impaired perfusion, oliguria, and late hypotension; edema may coexist.	Bland sediment, hemoconcentration, and low urine sodium may support the diagnosis but are nonspecific. A collapsible IVC may suggest low filling but is not diagnostic.
Volume-expanded/congestion- or pressure-associated	Sodium retention and extracellular volume expansion, with possible pressure-related reduction in filtration.	Active relapse, excess fluid or albumin, diuretic resistance, or positive fluid balance. Weight gain, hypertension, tense edema, ascites, or respiratory compromise.	Urine sodium may remain low despite expansion. Lung B-lines, POCUS, venous Doppler, or bioimpedance may help, but pediatric validation is limited.
Acute tubular stress/injury	Tubular injury from sustained ischemia, inflammation, nephrotoxins, or protein overload.	Prolonged hypoperfusion, sepsis, nephrotoxic exposure, or persistent proteinuria. AKI continues despite restored perfusion.	Granular casts or tubular cells may occur. NGAL and KIM-1 can increase, but proteinuria may elevate both. Ultrasound is usually nonspecific.
Infection- or sepsis-associated	Inflammation, microcirculatory dysfunction, altered perfusion, capillary leak, and tubular stress.	Peritonitis, pneumonia, cellulitis, urinary infection, bacteremia, or gastroenteritis with impaired perfusion or fluid overload.	Cultures and source-directed tests are central. Inflammatory markers are nonspecific; imaging should target the suspected source and hemodynamic state.
Drug-associated AKI/AIN	Hemodynamic GFR reduction, tubular toxicity, AIN, CNI vasoconstriction, or endothelial injury.	NSAIDs, RAAS blockers, antibiotics, CNIs, or cumulative nephrotoxic exposure. Temporal association is important; classic AIN features are often absent.	Urinary findings and eosinophilia are insensitive. Review drug levels and interactions; biopsy may be required in persistent diagnostic uncertainty.
Vascular/thrombotic	Renal vascular obstruction, systemic thrombosis, or microangiopathic endothelial injury.	Severe hypoalbuminemia, dehydration, infection, catheters, immobility, or thrombophilia. Abrupt AKI, pain, hematuria, asymmetric edema, or TMA features.	Hemolysis and coagulation studies are selected according to presentation. Urgent Doppler, followed by CT or MR vascular imaging when necessary.
Underlying glomerular disease/SRNS/FSGS or mimic	Severe glomerular injury, SRNS/FSGS, proliferative disease, or genetic/systemic disease.	Steroid resistance, delayed remission, persistent hypertension or proteinuria, active sediment, extrarenal features, or sustained kidney dysfunction.	Hematuria, red-cell casts, complement or serologic abnormalities may support intrinsic disease. Biopsy or genetic testing may be decisive.
Mixed or evolving	Mechanisms coexist or change over time; hemodynamic dysfunction may progress to tubular injury.	Discordant findings or an unexpected response to treatment, particularly during infection, nephrotoxic exposure, or changing fluid balance.	Serial clinical, laboratory, and POCUS reassessment is essential to detect phenotype evolution and avoid diagnostic anchoring.

Phenotypes are not mutually exclusive. No single clinical sign, urine index, biomarker, or POCUS measurement establishes the mechanism, so findings require serial, integrated interpretation. Key supporting sources: low-volume phenotype [5,6,28,30]; volume-expanded and pressure-associated phenotype [8,18,32]; tubular injury [7,24,33]; infection and sepsis [34]; drug-associated AKI and AIN [36,37,47]; vascular and thrombotic disease [39]; and underlying glomerular disease [43,44]. AKI, acute kidney injury; IVC, inferior vena cava; POCUS, point-of-care ultrasonography; NGAL, neutrophil gelatinase-associated lipocalin; KIM-1, kidney injury molecule-1; AIN, acute interstitial nephritis; GFR, glomerular filtration rate; CNI, calcineurin inhibitor; NSAIDs, nonsteroidal anti-inflammatory drugs; RAAS, renin–angiotensin–aldosterone system; TMA, thrombotic microangiopathy; CT, computed tomography; MR, magnetic resonance; SRNS, steroid-resistant nephrotic syndrome; FSGS, focal segmental glomerulosclerosis.

**Table 2:** Conventional and Emerging Biomarkers in Childhood Nephrotic Syndrome-Associated AKI.

Biomarker and Domain	Specimen and Timing	Potential Clinical Contribution	NS-Specific Limitations, Pediatric Evidence, and Readiness
Serum creatinine Functional filtration marker	Serum; baseline and serial values; delayed during non-steady state.	Defines AKI, stage, and recovery trajectory but not mechanism.	Influenced by age, muscle mass, dilution, baseline uncertainty, and kinetics. Routine/required, but inadequate for mechanistic phenotyping.
Urine output Functional/physiological marker	Timed or continuous measurement relative to weight and fluid balance.	May precede creatinine elevation and informs staging and therapeutic response.	Affected by collection accuracy, intake, diuretics, edema, and obstruction. Routine/required when measured reliably.
Serum cystatin C Functional filtration marker	Serum; baseline and serial values interpreted with steroid exposure.	May detect GFR change earlier than creatinine and is less muscle-dependent.	Influenced by corticosteroids, inflammation, thyroid status, age, and assay. Pediatric evidence is supportive but NS-specific data are limited. Adjunctive.
NGAL Tubular damage/inflammatory-response marker	Urine or plasma; early and serial measurements are timing-dependent.	May indicate tubular stress or injury before or alongside functional decline.	Influenced by proteinuria, infection, inflammation, CKD, disease activity, age, and sex. Direct pediatric NS-AKI evidence is limited. Investigational/adjunctive; no validated NS cutoff.
KIM-1 Proximal tubular damage marker	Urine; early or serial measurement with documented normalization method.	May support proximal tubular injury when combined with urine microscopy and other markers.	Influenced by protein overload, persistent proteinuria, chronic tubular injury, urine concentration, and age. Direct NS-AKI evidence is limited. Investigational.
[TIMP-2]·[IGFBP7] Tubular cellular-stress marker	Urine; timing relative to exposure and AKI is critical.	May identify cellular stress before overt functional deterioration but cannot determine etiology.	No NS-specific validation; results may vary with proteinuria, inflammation, timing, normalization, and assay. Pediatric evidence is heterogeneous. Investigational; no validated cutoff or management pathway.

Key supporting sources: creatinine and urine output [10]; cystatin C [50,51,52]; NGAL [24,33,53,60]; KIM-1 [24,33,55,56]; and [TIMP-2]·[IGFBP7] [57,58,59]. AKI, acute kidney injury; CKD, chronic kidney disease; GFR, glomerular filtration rate; IGFBP7, insulin-like growth factor-binding protein 7; KIM-1, kidney injury molecule-1; NGAL, neutrophil gelatinase-associated lipocalin; NS, nephrotic syndrome; TIMP-2, tissue inhibitor of metalloproteinases-2.

**Table 3:** Conceptual Functional and Structural AKI Matrix.

Functional Markers	Stress/Damage Markers	Possible Interpretation	Essential Caution
No functional change	No signal	No biochemical AKI detected at that time.	Very early injury or suboptimal timing remains possible.
Functional deterioration	No signal	Predominantly functional/hemodynamic AKI is possible.	Not proof of reversible “prerenal” AKI; evolving injury may be missed.
No functional change	Signal detected	Subclinical tubular stress/injury or non-AKI elevation.	Reassess proteinuria, infection, inflammation, chronic injury, and assay limits.
Functional deterioration	Signal detected	Functional AKI with concomitant tubular stress/injury is more likely.	Does not establish cause; integrate clinical phenotype and serial response.

This matrix adapts the ADQI functional and damage framework and is conceptual rather than a validated nephrotic-syndrome algorithm [9]. No pediatric nephrotic-syndrome-specific thresholds for the proposed stress or damage biomarkers have been derived and externally validated. The matrix is therefore intended primarily for research stratification and conceptual assessment and should not be used as a stand-alone basis for bedside treatment decisions. Biomarker findings must be interpreted serially and within the clinical phenotype. Functional deterioration refers to a meaningful change in serum creatinine, urine output, or potentially cystatin C, while a stress or damage signal refers to an elevation in markers such as NGAL, KIM-1, or [TIMP-2]·[IGFBP7]. ADQI, Acute Disease Quality Initiative; AKI, acute kidney injury; IGFBP7, insulin-like growth factor-binding protein 7; KIM-1, kidney injury molecule-1; NGAL, neutrophil gelatinase-associated lipocalin; TIMP-2, tissue inhibitor of metalloproteinases-2.

**Table 4:** From Phenotype to Management.

Phenotype	Initial Approach	Reassessment	Main Caution and Escalation
Low effective arterial volume/hemodynamic	Withhold diuretics and RAAS blockers; cautiously replace documented losses or treat shock. Consider albumin only with convincing underfilling.	Perfusion, blood pressure, urine output, fluid balance, weight, respiratory status, and creatinine.	Edema does not exclude underfilling, but hypoalbuminemia alone does not justify albumin. Escalate persistent shock to intensive care.
Volume-expanded/congestion- or pressure-associated	Avoid reflex fluids or albumin; restrict sodium, individualize fluids, and use appropriately dosed loop diuretics.	Weight, fluid balance, edema, blood pressure, oxygen requirement, urine output, electrolytes, and creatinine.	Low urine sodium does not prove underfilling. Avoid excessive decongestion; consider KRT for refractory fluid overload with organ compromise.
Acute tubular stress/injury	Provide supportive AKI care; remove nephrotoxins; maintain adequate perfusion; manage fluids, electrolytes, and acid-base balance.	Creatinine, urine output, fluid balance, potassium, bicarbonate, and uremic manifestations.	Do not force diuresis or repeat ineffective fluid challenges. Escalate for refractory AKI complications.
Infection- or sepsis-associated	Obtain cultures when feasible, start appropriate antimicrobials promptly, control the source, reassess immunosuppression, and provide phenotype-sensitive hemodynamic support.	Perfusion, blood pressure, respiratory status, cultures, fluid balance, urine output, and creatinine.	Avoid delayed antibiotics and indiscriminate fluids. Seek early critical-care support for shock or progressive AKI.
Drug-associated AKI/AIN	Withdraw suspected agents; assess interactions, CNI levels, blood pressure, potassium, and TMA features. Consider biopsy or corticosteroids selectively.	Kidney function after withdrawal, urine output, drug levels, potassium, blood count, and hemolysis indices.	Therapeutic CNI levels do not exclude toxicity; AIN findings are insensitive. Exclude active infection before corticosteroids.
Vascular/thrombotic	Obtain urgent site-directed imaging and initiate specialist-directed anticoagulation or intervention.	Kidney function, thrombus extent, imaging response, platelets, hemolysis, and bleeding.	Avoid delayed imaging or indiscriminate prophylaxis. Extensive bilateral disease or TMA may require intervention and KRT.
Underlying glomerular disease/SRNS/FSGS or mimic	Reassess diagnosis and disease activity using urine sediment, serology, kidney biopsy, and/or genetic testing; provide disease-specific treatment.	Proteinuria, blood pressure, sediment, serology, creatinine, and extrarenal features.	Avoid repeated volume treatment or unsupported immunosuppression. Rapidly progressive disease requires urgent specialist evaluation.
Mixed or evolving	Treat immediate threats using staged, reversible interventions and revise the phenotype serially.	Clinical and laboratory response after each intervention.	Avoid diagnostic anchoring and simultaneous opposing treatments. Escalate according to cumulative organ dysfunction.

This is a mechanism-based framework, not a validated treatment algorithm. Key supporting sources: universal AKI care [10,26]; low-volume and albumin strategy [62,63]; volume-expanded and diuretic strategy [6,19]; infection and sepsis [34,64]; drug-associated AKI and AIN [37,47]; vascular and thrombotic management [39]; SRNS and intrinsic disease [43]; and KRT [10,66]. AIN, acute interstitial nephritis; AKI, acute kidney injury; CNI, calcineurin inhibitor; FSGS, focal segmental glomerulosclerosis; KRT, kidney replacement therapy; RAAS, renin–angiotensin–aldosterone system; SRNS, steroid-resistant nephrotic syndrome; TMA, thrombotic microangiopathy.

## Data Availability

No new data were created or analyzed in this study. Data sharing is not applicable to this article.

## Abbreviations

The following abbreviations are used in this manuscript:

ADQI	Acute Disease Quality Initiative
AIN	Acute interstitial nephritis
AKD	Acute kidney disease
AKI	Acute kidney injury
CKD	Chronic kidney disease
CNI	Calcineurin inhibitor
CT	Computed tomography
eGFR	Estimated glomerular filtration rate
FSGS	Focal segmental glomerulosclerosis
GFR	Glomerular filtration rate
IGFBP7	Insulin-like growth factor-binding protein 7
IVC	Inferior vena cava
KDIGO	Kidney Disease: Improving Global Outcomes
KIM-1	Kidney injury molecule-1
KRT	Kidney replacement therapy
MR	Magnetic resonance
NGAL	Neutrophil gelatinase-associated lipocalin
NS	Nephrotic syndrome
NSAIDs	Nonsteroidal anti-inflammatory drugs
NT-proBNP	N-terminal pro-B-type natriuretic peptide
POCUS	Point-of-care ultrasonography
pRIFLE	Pediatric Risk, Injury, Failure, Loss, and End-stage kidney disease criteria
PRISMA	Preferred Reporting Items for Systematic Reviews and Meta-Analyses
RAAS	Renin-angiotensin-aldosterone system
SRNS	Steroid-resistant nephrotic syndrome
TIMP-2	Tissue inhibitor of metalloproteinases-2
TMA	Thrombotic microangiopathy
VExUS	Venous excess ultrasound
